# The Protective Effect of a Functional Food Consisting of *Astragalus membranaceus*, *Trichosanthes kirilowii*, and *Angelica gigas* or Its Active Component Formononetin against Inflammatory Skin Disorders through Suppression of TSLP via MDM2/HIF1α Signaling Pathways

**DOI:** 10.3390/foods12020276

**Published:** 2023-01-06

**Authors:** Na-Ra Han, Hi-Joon Park, Seong-Gyu Ko, Phil-Dong Moon

**Affiliations:** 1 College of Korean Medicine, Kyung Hee University, Seoul 02447, Republic of Korea; 2 Korean Medicine-Based Drug Repositioning Cancer Research Center, College of Korean Medicine, Kyung Hee University, Seoul 02447, Republic of Korea; 3 Department of Anatomy & Information Sciences, College of Korean Medicine, Kyung Hee University, Seoul 02447, Republic of Korea; 4 Department of Preventive Medicine, College of Korean Medicine, Kyung Hee University, Seoul 02447, Republic of Korea; 5 Center for Converging Humanities, Kyung Hee University, Seoul 02447, Republic of Korea

**Keywords:** JRP-SNF102, formononetin, functional food, human mast cell line-1, inflammation, thymic stromal lymphopoietin, ear edema

## Abstract

An herbal mixture (SH003) of *Astragalus membranaceus*, *Trichosanthes kirilowii*, and *Angelica gigas* exhibits therapeutic effects on carcinomas and immunosuppression. However, the role of JRP-SNF102, which is an advanced mixture of SH003, in regulating inflammatory responses is unexplored. We aim to substantiate the therapeutic potential of JRP-SNF102 and its active component, formononetin (FMN), as a functional food that moderates inflammatory responses. The inhibitory effects of JRP-SNF102 or FMN on thymic stromal lymphopoietin (TSLP) levels were evaluated in phorbol 12-myristate 13-acetate (PMA) plus A23187-activated human mast cell line-1 (HMC-1) cells and a mouse model of PMA-induced ear edema. The JRP-SNF102 or FMN inhibited the secretion and mRNA expression of TSLP and vascular endothelial growth factor (VEGF) in the activated HMC-1 cells. The expression levels of murine double minute 2 (MDM2), hypoxia-inducible factor 1α (HIF1α), and NF-κB were also suppressed by JRP-SNF102 or FMN in the activated HMC-1 cells. The JRP-SNF102 or FMN inhibited TSLP and VEGF levels, attenuating redness and ear thickness in mice with acute ear edema; JRP-SNF102 or FMN reduced the expression levels of MDM2, HIF1α, and NF-κB in the ear tissues. These findings suggest the potential for JRP-SNF102 as a functional food in the treatment of inflammatory skin disorders through suppression of TSLP and VEGF.

## 1. Introduction

Inflammation is the most common disease and is involved in numerous reactions such as wounds, redness, swelling, cancer, or arthritis [1]. Mast cells play important roles in the pathophysiology of inflammation, such as in the case of ear edema [2,3]. When mast cells are activated, they are recruited to the site of inflammation in large numbers [4]. In ear skin inflammation, they induce ear edema [5]. The activated mast cells release various inflammatory cytokines, including thymic stromal lymphopoietin (TSLP) [6]. TSLP is an important upstream cytokine that induces type 2 inflammation in ear skin inflammation [7,8,9].

Previous studies have reported that *Astragalus membranaceus* (Fisch.) Bunge (family: Fabaceae), *Trichosanthes kirilowii* (Maxim.) (family: Cucurbitaceae), and *Angelica gigas Nakai* (family: Apiaceae) are useful as functional foods [10,11,12]. An herbal mixture of *Astragalus membranaceus*, *Trichosanthes kirilowii*, and *Angelica gigas* in a ratio of 1:1:1, known as SH003, has been reported to exhibit anticarcinogenic activity in breast cancer [13], lung cancer [14], cervical cancer [15], and gastric cancer [16], as well as benefits against neuropathic pain [17,18] and immune-enhancing [19] effects in vitro and in vivo. JRP-SNF102, an advanced mixture of SH003, consists of *Astragalus membranaceus*, *Trichosanthes kirilowii,* and *Angelica gigas* in a ratio of 3:1:1. *Astragalus membranaceus* was reported to show anti-tumor, immunomodulatory, and anti-viral effects in vitro and in vivo [20]. *Trichosanthes kirilowii* was determined to have anti-tumor and immunomodulatory effects in vitro and in vivo [21,22]. *Angelica gigas* was reported to ameliorate cognitive impairment or neuroinflammation in in vivo models [23,24]. Formononetin (FMN) is an active component of *Astragalus membranaceus* [25], which has been reported to have pharmacological properties, such as being able to mitigate allergic inflammatory reactions, diabetic nephropathy, or tumors [26,27,28]. Interestingly, several anti-cancer agents have been reported to have anti-inflammatory effects [29,30,31,32,33]. In addition, inflammation is an important component of cancer progression and neuropathic pain [18,34]. Many studies have reported that SH003 exerts beneficial effects on cancers and neuropathic pain [13,14,15,16,17,18]. Thus, we speculated that JRP-SNF102 would be effective in its inflammatory responses because JRP-SNF102 has the same composition as SH003, albeit with changed proportions. The aim of this study is therefore to determine the therapeutic potential of JRP-SNF102 and FMN as a functional food in inflammatory responses. We studied the immunoregulatory effects of JRP-SNF102 and FMN on mast cell-derived TSLP, discovered a molecular mechanism regulating the protective effects, and verified the effects using an animal model of ear edema.

## 2. Materials and Methods

### 2.1. Preparation

**Figure 1:**
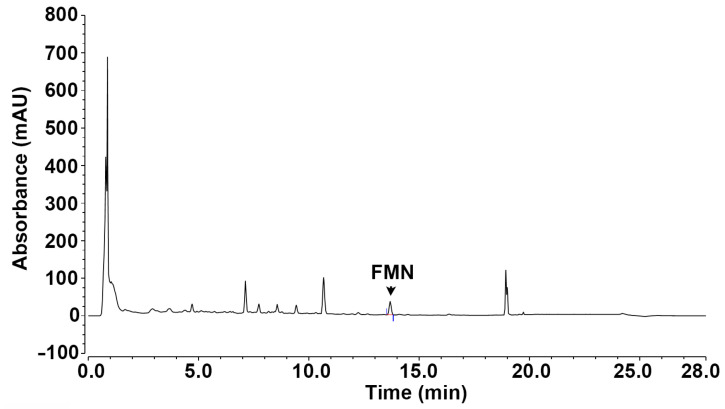
FMN identified in JRP-SNF102 by HPLC.

The JRP-SNF102 (Jaein R&P, Seoul, Republic of Korea) was prepared by referring to previous work [19]. *Astragalus membranaceus*, *Trichosanthes kirilowii*, and *Angelica gigas* were each extracted in 30% ethanol and the resulting freeze-dried powder was again dissolved in 30% ethanol in a ratio of 3:1:1 to prepare 100 mg/mL. The JRP-SNF102 was identified and standardized by marker compounds in high performance liquid chromatography (HPLC) analysis based on our previous studies (Figure 1) [19,35]. The FMN (purity ≥ 99%; Sigma Chemical Co., St. Louis, MO, USA) and dexamethasone (Dex, Sigma Chemical Co.) were dissolved in DMSO (0.01%) [36,37].

### 2.2. Cell Culture and Treatment

Human mast cell line-1 (HMC-1) cells were incubated in Iscove’s Modified Dulbecco’s Medium, which was supplemented with 10% heat-inactivated fetal bovine serum, 100 IU/mL penicillin, and 100 µg/mL streptomycin (Gibco BRL, Grand Island, NY, USA). HMC-1 cells were activated by phorbol 12-myristate 13-acetate (PMA, 50 nM) and calcium ionophore A23187 (1 μM) in the presence or absence of JRP-SNF102 (4, 40, and 400 µg/mL), FMN (10 µM), or Dex (100 nM).

### 2.3. Cell Viability

Cell viability analysis was conducted as previously described [19].

### 2.4. ELISA

Human/mouse TSLP (R&D Systems, Minneapolis, MN, USA) and human/mouse vascular endothelial growth factor (VEGF, R&D Systems) were measured in cells supernatants, ear tissue homogenate, or serum using enzyme-linked immunosorbent assay (ELISA).

### 2.5. qPCR

Total RNA was extracted with an easy-BLUE™ RNA extraction kit (iNtRON Biotech Inc., Seongnam, Republic of Korea). The cDNA was generated by a cDNA reverse transcription kit (Bioneer Corporation, Daejeon, Republic of Korea). Quantitative real-time PCR was run with Power SYBR^®^ Green Master Mix (Thermo Fisher Scientific, Sunnyvale, CA, USA) with each primer (Table 1). The fold changes of target genes were normalized to GAPDH by a ΔΔCt method.

### 2.6. Western Blotting Analysis

Western blotting analysis was conducted as previously described [19] using primary antibodies against MDM2, actin, HIF1α, histone, NF-κB, and pIkBα, and then the second antibodies (Santa Cruz Biotechnology, Santa Cruz, CA, USA).

### 2.7. Analysis Immunofluorescence Staining

Immunofluorescence staining was conducted as previously described [38,39] using primary antibodies against c-kit (Santa Cruz Biotechnology) and TSLP (R&D System, Inc.) and secondary antibodies conjugated to rat IgG-fluorescein isothiocyanate antibody, and mouse IgG-tetramethylrhodamine isothiocyanate antibodies (Abcam, Cambridge, MA, USA).

### 2.8. Animal Studies

Animal studies were approved by the Animal Care Committee of Kyung Hee University (No. KHSASP-20-472) and performed according to the US guidelines (NIH publication, no. 85-23, revised in 1985). ICR mice (male, 4 weeks, Dae-Han Experimental Animal Center, Eumseong, Republic of Korea) were randomly divided into five groups: normal group (acetone-applied mice), PMA-challenged control group (PMA-challenged mice), JRP-SNF102 (400 mg/kg)-treated groups with PMA challenge, FMN (10 µM)-treated groups with PMA challenge, and Dex (100 nM)-treated groups with PMA challenge. Six mice were used per group. The ear edema study was conducted as previously described [38,40]. The ear thickness of mice orally administered with PBS, JRP-SNF102, FMN, or Dex was measured with a micrometer after anesthesia. A mixture of Zoletil^®^50 (Virbac, Carros, France) and Rompun^®^ (Bayer HealthCare, Leverkusen, Germany) in a ratio of 1:1 was used to anesthetize the mice (1 µL/g, i.p.). In the PMA-challenged mice, topical PMA treatment (20 μL of 0.1 mg/mL PMA solution in acetone) was given to both the inner and outer surfaces of the ear, while the normal group was given an equal dose and volume of acetone. The ear thickness was evaluated 6 h after the PMA challenge in the anesthetized mice. The cardiac blood was removed at the end of the animal studies. All mice were subsequently euthanized via CO_2_ inhalation.

### 2.9. HPLC Analysis

Chromatographic analysis was performed to identify and quantify FMN in JRP-SNF102 using ultra-HPLC (UHPLC, Vanquish, Thermo Fisher Scientific, Sunnyvale, CA, USA) equipped with an ACQUTY UPLC HSS T3 column (2.1 mm × 100 mm, 1.8 μm; waters). The mobile phase for 0.1% formic acid in water (eluent A) and 0.1% formic acid in acetonitrile (eluent B) was under the following gradient profile: 0–1 min; 5% B, 1–4 min; 5–15% B, 4–11 min; 15–35% B, 11–17 min; 35–50% B, 17–19 min; 50–100% B, 19–23 min; 100% B and equilibration with 5% B for 4 min. The flow rate was 0.4 mL/min. The injection volume was 5 μL. The column temperature was maintained at 40 °C. The eluents were monitored using a diode array detector at 260 nm. The content of FMN in the JRP-SNF102 was 241 ± 0.003 μg/g (retention time, 13.69 min).

### 2.10. Statistical Analyses

All the experimental data are shown as mean ± SEM. SPSS statistics software was used for all statistical analyses (SPSS Inc., Chicago, IL, USA). Significant differences were assessed by one-way ANOVA followed by post hoc Tukey’s test. The *p*-value < 0.05 was accepted as statistically significant.

## 3. Results

### 3.1. JRP-SNF102 or FMN Inhibited TSLP Secretion in Activated HMC-1 Cells

**Figure 2:**
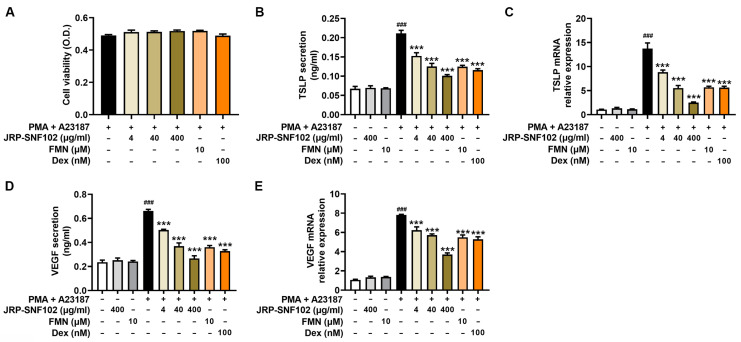
An inhibitory effect of JRP-SNF102 or FMN on TSLP secretion. HMC-1 cells were incubated with or without JRP-SNF102, FMN, or Dex for 1 h, followed by PMA and A23187 stimulating for 7 h. (**A**) The cell viability by an MTT assay. (**B**,**D**) The secretion of TSLP and VEGF by ELISA. (**C**,**E**) HMC-1 cells were incubated with or without JRP-SNF102, FMN, or Dex for 1 h, followed by PMA and A23187 stimulating for 5 h. The mRNA levels of TSLP and VEGF by qPCR. ^###^ *p <* 0.001 versus normal group; *** *p <* 0.001 versus PMA and A23187-stimulated group. −, non-treatment; +, treatment.

To investigate the potential inhibitory effects of JRP-SNF102 and FMN on TSLP secretion from mast cells, PMA and A23187-stimulated HMC-1 cells were used. Pretreatment with JRP-SNF102 showed no obvious cytotoxicity in the activated HMC-1 cells over a range of concentrations (4–400 μg/mL; Figure 2A, Supplementary Tables S1 and S2). FMN (10 μM) showed no obvious cytotoxicity in the activated HMC-1 cells (Figure 2A, Supplementary Tables S1 and S2). These doses were also determined by reference to previous studies [19,36]. Notably, the JRP-SNF102 dose dependently decreased the TSLP secretion induced by PMA and A23187 (*p* < 0.05, Figure 2B, Supplementary Tables S1 and S3). The mRNA expression of TSLP was reduced in the JRP-SNF102-treated and activated HMC-1 cells (*p <* 0.05, Figure 2C, Supplementary Tables S1 and S4). The FMN showed inhibitory effects on the secretion and mRNA expression of TSLP (*p <* 0.05, Figure 2B,C, Supplementary Tables S1, S3 and S4). JRP-SNF102 (4, 40, and 400 μg/mL) or FMN suppressed the secretion and mRNA expression of VEGF (*p <* 0.05, Figure 2D,E, Supplementary Tables S1, S5 and S6). Dex, used as a positive control [38], showed the inhibitory effects on these levels (*p <* 0.05, Figure 2, Supplementary Tables S1–S6).

### 3.2. JRP-SNF102 or FMN Suppressed MDM2 Expression in Activated HMC-1 Cells

**Figure 3:**
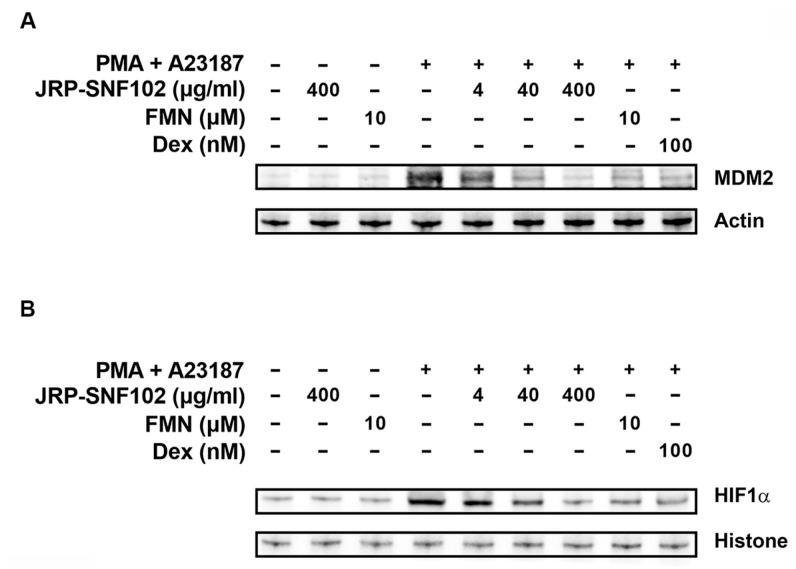
An inhibitory effect of JRP-SNF102 or FMN on MDM2 expression. HMC-1 cells were incubated with or without JRP-SNF102, FMN, or Dex for 1 h, followed by PMA and A23187 stimulating for 3 h. (**A**) MDM2 level was determined by Western blotting. (**B**) HMC-1 cells were incubated with or without JRP-SNF102, FMN, or Dex for 1 h, followed by PMA and A23187 stimulating for 4 h. HIF1α level in nucleus was determined by Western blotting. −, non-treatment; +, treatment.

The MDM2 pathway is known to be a major inflammatory signaling pathway, enhancing inflammatory responses by up-regulating inflammatory mediators [41]. Our previous work reported that MDM2 regulates TSLP and VEGF levels in activated HMC-1 cells [38]. Thus, we explored whether JRP-SNF102 and FMN regulates MDM2 expression in activated HMC-1 cells. As expected, immunoblot images showed a downward trend in the expression level of MDM2, after treatment with JRP-SNF102 or FMN (Figure 3A). As a downstream mediator of MDM2, HIF1α regulates the inflammatory responses, increasing the production of TSLP and VEGF [38,42]. Figure 3B shows that JRP-SNF102 or FMN suppressed the nuclear HIF1α expression levels increased by PMA and A23187. Dex also indicated the inhibitory effects of MDM2 and HIF1α levels (Figure 3).

### 3.3. JRP-SNF102 or FMN Suppressed Nuclear NF-κB Expression in Activated HMC-1 Cells

**Figure 4:**
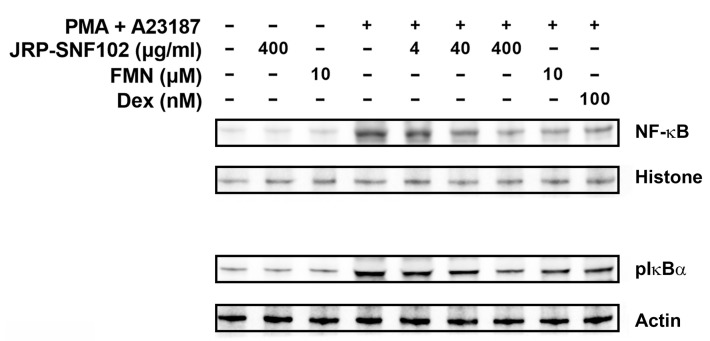
An inhibitory effect of JRP-SNF102 or FMN on NF-κB expression. HMC-1 cells were incubated with or without JRP-SNF102, FMN, or Dex for 1 h, followed by PMA and A23187 stimulating for 2 h. NF-κB level in nucleus and pIkBα level in cytoplasm were determined by Western blotting. −, non-treatment; +, treatment.

In addition to MDM2/HIF1α pathways, to determine whether JRP-SNF102 and FMN could act as a regulator of NF-κB expression in mast cells, we further investigated the regulatory effects of JRP-SNF102 and FMN on the nuclear NF-κB expression in the activated HMC-1 cells. The PMA and A23187 resulted in an increase in the expression levels of NF-κB in the nucleus of HMC-1 cells, while JRP-SNF102 or FMN suppressed the expression levels (Figure 4). The JRP-SNF102 or FMN also decreased the expression levels of pIkBα increased by PMA and A23187 in cytoplasm (Figure 4). These results indicate that JRP-SNF102 and FMN prevents TSLP and VEGF secretion from the activated HMC-1 cells by modulating MDM2/HIF1α and NF-κB signaling pathways.

### 3.4. JRP-SNF102 or FMN Suppressed PMA-Induced Ear Edema

**Figure 5:**
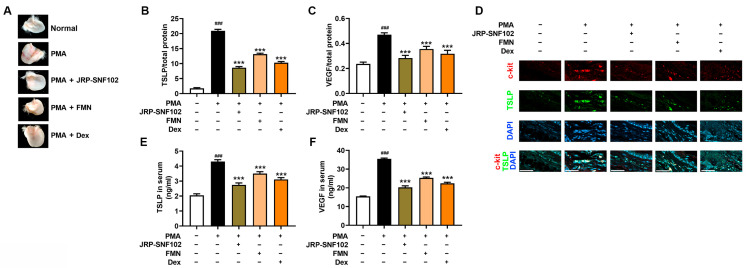
A suppressive effect of JRP-SNF102 or FMN on TSLP levels in PMA-induced ear edema. (**A**) The photographs of ear biopsies were obtained after ear thickness analysis. The ear biopsies were prepared for determining (**B**) TSLP and (**C**) VEGF levels by ELISA. (**D**) In the ear biopsies, c-kit (+) and TSLP (+) cells were observed with a confocal microscope, scale bar = 50 μm. (**E**) TSLP and (**F**) VEGF levels in serum samples were quantified by ELISA. ^###^ *p <* 0.001 versus normal group; *** *p <* 0.001 versus PMA-challenged groups. *n* = 6. −, non-treatment; +, treatment.

**Figure 6:**
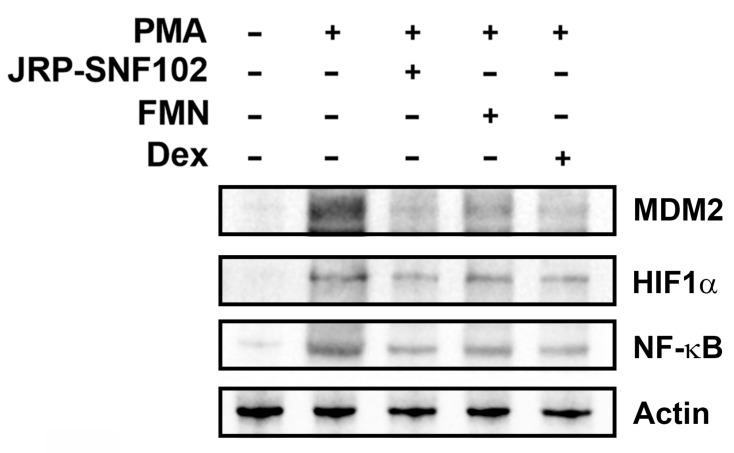
A suppressive effect of JRP-SNF102 or FMN on MDM2, HIF1α, and NF-κB levels in PMA-induced ear edema. The MDM2, HIF1α, and NF-κB levels were determined by Western blotting. −, non-treatment; +, treatment.

There is much evidence that PMA-induced ear edema is accompanied by inflammatory responses [43]. As presented in Figure 5A and Table 2, PMA irritation caused redness and increased ear thickness. The JRP-SNF102 or FMN treatment remarkably reversed the redness and the increased ear thickness (*p <* 0.05, Figure 5A and Table 2). Moreover, the expression levels of TSLP and VEGF in the ear tissue biopsy were significantly reduced by JRP-SNF102 or FMN (*p <* 0.05, Figure 5B,C). Consistent with these findings, histological analysis showed that JRP-SNF102 or FMN decreased the mast cell-derived TSLP expression in the ear biopsy (Figure 5D). Similarly, JRP-SNF102 or FMN also significantly reduced the levels of TSLP and VEGF in serum (*p <* 0.05, Figure 5E,F). As JRP-SNF102 or FMN was found to inhibit MDM2, HIF1α, and NF-κB levels in vitro, the features were investigated in the ear tissue biopsy. Immunoblotting showed that the MDM2, HIF1α, and NF-κB levels were elevated by the PMA challenge, indicating these changes were reduced by JRP-SNF102 or FMN treatment (Figure 6).

## 4. Discussion

This study identified that JRP-SNF102 and FMN have a beneficial effect on inflammatory responses. In vitro experiments showed that JRP-SNF102 and FMN effectively suppressed TSLP and VEGF levels via down-regulation of MDM2/HIF1α and NF-κB pathways in activated mast cells. This effect was verified in an in vivo model of ear edema by demonstrating reductions in TSLP, VEGF, MDM2, HIF1α, and NF-κB levels in the serum or ear tissues, along with reductions in redness and ear thickness by the treatment with JRP-SNF102 and FMN. These findings reveal that JRP-SNF102 could be applied to the treatment of inflammatory skin disorders, suggesting the possibility of treating a variety of inflammatory responses.

TSLP and VEGF released from activated mast cells were reported to contribute to tissue inflammation [6,44,45]. In inflammatory skin disorders and airway inflammation in in vivo models, TSLP was highly expressed [46]. TSLPR-knockout mice exhibited a severely attenuated lung inflammation with less infiltration of inflammatory cells [47]. The lungs of transgenic mice overexpressing TSLP showed massive infiltration of inflammatory cells and airway inflammatory disease [48]. An anti-VEGF therapy decreased retinal thickening and inflammatory responses in individuals with eyes edema [49]. Several studies reported that *Astragalus membranaceus* inhibited TSLP levels in epithelial cells and a murine model of allergic contact dermatitis and had a therapeutic effect on allergic diseases [50,51]. Ku et al. [52] found that an herbal mixture including *Angelica gigas* decreased TSLP mRNA levels in mast cells, indicating that it might be useful for treating atopic dermatitis. FMN was reported to inhibit TSLP levels in keratinocytes and mouse models of atopic dermatitis [36,53]. It was reported that FMN inhibited VEGF levels in epithelial cells and to alleviate the diabetic retinopathy in rats [25]. Furthermore, the present study showed that JRP-SNF102 and FMN remarkably decreased the TSLP and VEGF levels in the activated mast cells and the ear tissue and serum of mice with acute ear edema, indicating that JRP-SNF102 can protect against inflammatory responses by inhibiting TSLP and VEGF levels.

The therapeutic targeting of mechanisms as well as mediators during inflammation reduced the number of recruited mast cells and attenuated the subsequent effects of mast cells activation in a location [4]. Several reports found that the MDM2/HIF1α and NF-κB pathways were implicated in the pathological process of inflammation as well as tumor [42,54]. Thomasova et al. [54] and Gu et al. [55] reported that the MDM2 activated the NF-κB signaling, which was involved in inflammatory responses. Crosstalk of HIF1α and NF-κB regulated critical inflammatory functions, which resulted in the expression levels of proinflammatory cytokines [42]. Moreover, our previous works found that the MDM2, HIF1α, and NF-κB levels were enhanced during various inflammatory responses [38,56,57]. Inhibition of MDM2 was shown to have promising therapeutic efficacy for treating inflammation in preclinical models [58]. In mice, HIF-1α deletion resulted in reduced pro-inflammatory cytokines and inflammatory responses [59]. Miyake et al. [60] mentioned that mast cell accumulation would be reduced by inhibition of NF-kB activation. *Astragalus membranaceus* was reported to reduce MDM2 levels in tumor cells and had an anti-tumor effect [61]. *Trichosanthes kirilowii* seeds were documented as showing inhibitory activity on tumor cell growth by limiting NF-κB or HIF1α in vitro and in vivo [62,63]. An active component of *Angelica gigas* was reported as having an anti-cancer effect by down-regulating the protein level of HIF-1α in vitro and in vivo [64]. Wu et al. [25] mentioned that FMN alleviated diabetic retinopathy through suppression of the HIF-1α/VEGF signaling pathway in vitro and in vivo. Furthermore, this current study showed that JRP-SNF102 and FMN markedly suppressed the levels of MDM2, HIF1α, and NF-κB in the activated mast cells and the ear tissue of mice with acute ear edema. This provides additional evidence supporting the therapeutic potential of JRP-SNF102 in various inflammatory reactions induced or exacerbated through MDM2/HIF1α and NF-κB pathways.

## 5. Conclusions

In conclusion, we found that JRP-SNF102 and FMN reduced TSLP release from mast cells through MDM2/HIF1α and NF-κB pathways. In vivo studies indicated that JRP-SNF102 and FMN ameliorated the mast cell-derived TSLP levels in ear edema. More importantly, we demonstrated the underlying mechanisms of JRP-SNF102 and FMN in suppressing the expression levels of MDM2, HIF1α, and NF-κB in in vitro and in vivo studies. Collectively, these findings may contribute to elucidating anti-inflammatory effects of JRP-SNF102 and offering scientific evidence for functional food applications of *Astragalus membranaceus*, *Trichosanthes kirilowii*, and *Angelica gigas* in mast cell-mediated inflammatory responses. However, further clinical applications of JRP-SNF102 require in-depth and systematic studies in various experimental models, such as in chronic inflammatory response.

## Figures and Tables

**Table 1:** The sequences of primers.

Target Gene	Forward	Reverse
TSLP	5′-CGC CAC AAT CCT TGT AAT TGT G-3′	5′-CCC AGG CTA TTC GGA AAC TCA G-3′
VEGF	5′-AGG CCC ACA GGG ATT TTC TT-3′	5′-ATC AAA CCT CAC CAA GGC CA-3′
GAPDH	5′-TCG ACA GTC AGC CGC ATC TTC TTT-3′	5′-ACC AAA TCC GTT GAC TCC GAC CTT-3′

**Table 2:** The increase of ear thickness.

Group	Pre-Thickness (mm)	Post-Thickness (mm)	Increase (mm)
Normal	0.3380 ± 0.0017	0.3447 ± 0.0022	0.0067 ± 0.0012
PMA	0.3367 ± 0.0020	0.4361 ± 0.0091	0.0995 ± 0.0075 ^#^
PMA + JRP-SNF102	0.3378 ± 0.0021	0.3818 ± 0.0048	0.0440 ± 0.0037 *
PMA + FMN	0.3385 ± 0.0019	0.4028 ± 0.0043	0.0643 ± 0.0026 *
PMA + Dex	0.3381 ± 0.0017	0.3948 ± 0.0052	0.0567 ± 0.0064 *

Normal, acetone-applied mice; PMA, PMA-challenged control group; PMA + JRP-SNF102, JRP-SNF102 (400 mg/kg)-treated groups with PMA challenge; PMA + FMN, FMN (10 µM)-treated groups with PMA challenge; PMA + Dex, Dex (100 nM)-treated groups with PMA challenge. ^#^ *p <* 0.05 versus normal group; * *p <* 0.05 versus PMA-challenged groups. *n* = 6.

## Data Availability

All data are included in this publication.

## Supplementary Materials

The following supporting information can be downloaded at: https://www.mdpi.com/article/10.3390/foods12020276/s1. Table S1: The one-way ANOVA of Figure 2; Table S2: The post-hoc Tukey’s test of Figure 2A; Table S3: The post-hoc Tukey’s test of Figure 2B; Table S4: The post-hoc Tukey’s test of Figure 2C; Table S5: The post-hoc Tukey’s test of Figure 2D; Table S6: The post-hoc Tukey’s test of Figure 2E.
