# Supplementary material for: The Protective Effect of a Functional Food Consisting of Astragalus membranaceus, Trichosanthes kirilowii, and Angelica gigas or Its Active Component Formononetin against Inflammatory Skin Disorders through Suppression of TSLP via MDM2/HIF1α Signaling Pathways
Source: Foods. 2023 Jan 6;12(2):276. doi: 10.3390/foods12020276 (PMC9858287; doi:10.3390/foods12020276)
Supplement: Supplementary file 1 [file foods-12-00276-s001.zip › foods-2081216-supplementary.pdf]

Supplementary Table S1. The one-way ANOVA of Figure 2.

	***p*-value**	***F*-value**	**R^2**
**Figure 2A**	0.028	$F(5,30) = 2.946$	0.329
**Figure 2B**	0.000	$F(8,36) = 66.968$	0.937
**Figure 2C**	0.000	$F(8,36) = 77.808$	0.945
**Figure 2D**	0.000	$F(8,36) = 76.217$	0.944
**Figure 2E**	0.000	$F(8,36) = 174.323$	0.975

Supplementary Table S2. The post-hoc Tukey's test of Figure 2A.

	Cell viability (O.D.)	*p*-value
**PMA + A23187**	0.4907 ± 0.0052	-
**PMA + A23187 + JRP-SNF102 (4 μg/ml)**	0.5113 ± 0.0119	0.432
**PMA + A23187 + JRP-SNF102 (40 μg/ml)**	0.5130 ± 0.0058	0.347
**PMA + A23187 + JRP-SNF102 (400 μg/ml)**	0.5177 ± 0.0059	0.168
**PMA + A23187 + FMN (10 μM)**	0.5183 ± 0.0042	0.150
**PMA + A23187 + Dex (100 nM)**	0.4890 ± 0.0103	1.000

Supplementary Table S3. The post-hoc Tukey's test of Figure 2B.

	**TSLP secretion (ng/ml)**	***p*-value**
**Normal**	0.0673 ± 0.0062	-
**JRP-SNF102 (400 μg/ml)**	0.0697 ± 0.0053	1.000
**FMN (10 μM)**	0.0686 ± 0.0012	1.000
**PMA + A23187**	0.2114 ± 0.0078	0.000###
**PMA + A23187 + JRP-SNF102 (4 μg/ml)**	0.1527 ± 0.0083	0.000***
**PMA + A23187 + JRP-SNF102 (40 μg/ml)**	0.1253 ± 0.0079	0.000***
**PMA + A23187 + JRP-SNF102 (400 μg/ml)**	0.1009 ± 0.0032	0.000***
**PMA + A23187 + FMN (10 μM)**	0.1246 ± 0.0036	0.000***
**PMA + A23187 + Dex (100 nM)**	0.1159 ± 0.0037	0.000***

###$p < 0.001$ versus normal group; ***$p < 0.001$ versus PMA and A23187-stimulated group.

Supplementary Table S4. The post-hoc Tukey's test of Figure 2C.

	TSLP mRNA relative expression	*p*-value
**Normal**	1.0626 ± 0.0645	-
**JRP-SNF102 (400 μg/ml)**	1.3174 ± 0.1856	1.000
**FMN (10 μM)**	1.1246 ± 0.0614	1.000
**PMA + A23187**	13.7386 ± 1.1770	0.000###
**PMA + A23187 + JRP-SNF102 (4 μg/ml)**	8.8296 ± 0.4497	0.000***
**PMA + A23187 + JRP-SNF102 (40 μg/ml)**	5.5190 ± 0.5407	0.000***
**PMA + A23187 + JRP-SNF102 (400 μg/ml)**	2.5158 ± 0.1287	0.000***
**PMA + A23187 + FMN (10 μM)**	5.6846 ± 0.2271	0.000***
**PMA + A23187 + Dex (100 nM)**	5.6462 ± 0.2624	0.000***

###$p < 0.001$ versus normal group; ***$p < 0.001$ versus PMA and A23187-stimulated group.

Supplementary Table S5. The post-hoc Tukey's test of Figure 2D.

	**VEGF secretion (ng/ml)**	***p*-value**
**Normal**	0.2360 ± 0.0170	-
**JRP-SNF102 (400 μg/ml)**	0.2514 ± 0.0191	0.999
**FMN (10 μM)**	0.2419 ± 0.0080	1.000
**PMA + A23187**	0.6620 ± 0.0127	0.000###
**PMA + A23187 + JRP-SNF102 (4 μg/ml)**	0.5040 ± 0.0058	0.000***
**PMA + A23187 + JRP-SNF102 (40 μg/ml)**	0.3692 ± 0.0265	0.000***
**PMA + A23187 + JRP-SNF102 (400 μg/ml)**	0.2668 ± 0.0219	0.000***
**PMA + A23187 + FMN (10 μM)**	0.3606 ± 0.0135	0.000***
**PMA + A23187 + Dex (100 nM)**	0.3276 ± 0.0112	0.000***

###$p < 0.001$ versus normal group; ***$p < 0.001$ versus PMA and A23187-stimulated group.

Supplementary Table S6. The post-hoc Tukey's test of Figure 2E.

	VEGF mRNA relative expression	*p*-value
**Normal**	1.0664 ± 0.0657	-
**JRP-SNF102 (400 μg/ml)**	1.3288 ± 0.1182	0.984
**FMN (10 μM)**	1.3668 ± 0.0538	0.964
**PMA + A23187**	7.8316 ± 0.0476	0.000###
**PMA + A23187 + JRP-SNF102 (4 μg/ml)**	6.2320 ± 0.3486	0.000***
**PMA + A23187 + JRP-SNF102 (40 μg/ml)**	5.7198 ± 0.1254	0.000***
**PMA + A23187 + JRP-SNF102 (400 μg/ml)**	3.7054 ± 0.1693	0.000***
**PMA + A23187 + FMN (10 μM)**	5.4970 ± 0.2377	0.000***
**PMA + A23187 + Dex (100 nM)**	5.2868 ± 0.2630	0.000***

###$p < 0.001$ versus normal group; ***$p < 0.001$ versus PMA and A23187-stimulated group.
